# Clinical patterns of thalidomide in the treatment of transfusion-dependent β-thalassaemia in children: a prospective single-arm study in China

**DOI:** 10.1080/07853890.2025.2561219

**Published:** 2025-10-11

**Authors:** Xinyu Li, Weicong Hong, Jinni Wu, Qianyi Liu, Yong Liu, Shuting Hu, Luhong Xu, Honggui Xu, Danping Zhong, Junjiu Huang, Jianpei Fang

**Affiliations:** ^a^ Guangdong Provincial Key Laboratory of Malignant Tumor Epigenetics and Gene Regulation, Sun Yat-sen Memorial Hospital, Sun Yat-sen University, Guangzhou, China; ^b^ Department of Pediatrics, Sun Yat-sen Memorial Hospital, Sun Yat-sen University, Guangzhou, China; ^c^ MOE Key Laboratory of Gene Function and Regulation, State Key Laboratory of Biocontrol, School of Life Sciences, Sun Yat-sen University, Guangzhou, China; ^d^ Children’s Hospital of Shanghai, Shanghai Jiaotong University, Shanghai, China; ^e^ Follow-Up Center, Sun Yat-sen Memorial Hospital, Sun Yat-sen University, Guangzhou, China

**Keywords:** Transfusion dependent β-thalassaemia, thalidomide, children, growth velocity, neuropathy

## Abstract

**Background:**

Previous studies in β-thalassaemia have shown that thalidomide is effective in reducing transfusion volume and is safe for short-term therapy of 6 months or less. The aim of this prospective study was to investigate long-term benefits and side effects of thalidomide in the treatment of transfusion-dependent β-thalassaemia (TDT) in children.

**Methods:**

We analyzed the data from children and adolescents with TDT enrolled in a single-arm prospective study. The overall response rate, the incidence of discontinuation due to adverse events, the effect on height growth, and the effect on nerve conduction velocity were analyzed when patients received thalidomide for up to 1 year, or until unacceptable toxicity during the extended follow-up. The Kruskal-Wallis H test, one-way ANOVA, the Cochran-Mantel-Haenszel Chi-square test, and others were used for analysis.

**Results:**

The overall response rate was 82.9%. The most common grade 3–4 adverse events were abdominal pain, haemolysis, and leukocytopenia/neutropenia. Of the 73 patients who underwent nerve conduction velocity assessment during treatment for more than 12 months, 12 patients developed polyneuropathy and 26 developed motor neuropathy. The standard deviation scores (SDS) of height in major responders were significantly improved (−1.57, 95% CI (−1.95, −1.18)) at the end of 12 to 18-month treatment (*p* < 0.001). 64% of patients showed improvement in SDS of height.

**Conclusion:**

Thalidomide is associated with long-term benefits in growth and transfusion burden. A minority of patients required prolonged therapy beyond 6 months to achieve a response. Neuropathy and unexpected adverse events prevent the long-term use of thalidomide in children with thalassaemia and close monitoring is needed. (*chictr.org.cn identifiers: ChiCTR2000034998*)

## Introduction

Thalassaemia is a major health problem for millions of people. Although transfusions and iron chelation could improve the life expectancy of patients with transfusion-dependent thalassaemia (TDT), many of these patients do not have adequate access to the regular transfusion. Allogeneic haematopoietic stem cell transplantation is the curative therapy for patients with TDT, and many patients are waiting for suitable donors while taking inducer of foetal haemoglobin (HbF) expression to reduce the blood transfusion burden [1].

Thalidomide is one of the inducers of HbF expression [2], making it a potential treatment for β-thalassaemia [3–5]. In recent years, thalidomide has been recognized as an effective treatment for children with TDT [4,6–8], while serious side effects are uncommon with short-term therapy [4,9]. By achieving transfusion independence, patients also benefit in terms of iron overload, LDH levels, liver size, and spleen size as well [8,10].

However, numerous side effects of thalidomide, such as peripheral sensory neuropathy, deep vein thrombosis, and somnolence, have been reported in both adult and paediatric populations with other diseases [11–16]. Longitudinal patterns of neuropathy in children and adolescents with Crohn’s disease were reported by Liew, et al. [17], who suggested regular neurophysiological monitoring of those receiving thalidomide [17]. Evidence of the benefit and safety of thalidomide in the long-term therapy up to several years in children with TDT is still scarce [8,16,18–20]. We have previously reported the efficacy of thalidomide in inducing transfusion independence in children with TDT in a retrospective study [6]. The aim of this prospective study was to investigate long-term benefits and side effects.

## Methods

### Design, patients, and setting

This was a prospective, single-arm, longitudinal study. This study received approval from the ethics committee of Sun Yat-sen Memorial Hospital of Sun Yat-sen University (2019-KY-072). Informed consent was obtained from all participants and the parents/guardians of the children under 18 years of age. This study was conducted in accordance with the CONSORT guidelines.

**Figure 1.**
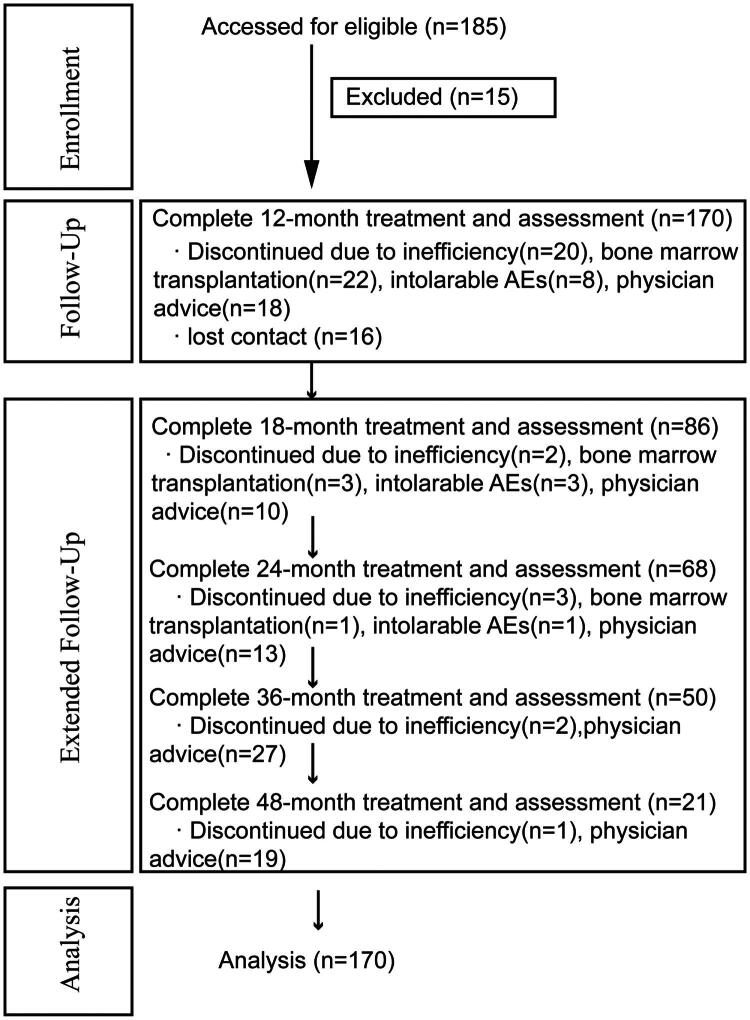
The flowchart of inclusion, follow-up and analysis.

Participants were Chinese TDT patients aged between 5 and 18 years, whose family members signed the informed consent, who had not participated in any other clinical trial in the previous 3 months, and who were not treated with drugs that interfere with therapeutic effects. The definition of TDT is a requirement for lifelong regular transfusion due to complexed β-globin gene mutations, either heterozygous or homozygous. Patients with hepatic, renal or cardiac dysfunction, other anaemia diseases, malignant tumours or rheumatic immune diseases were excluded from the outset. Exclusion/termination criteria were voluntary discontinuation of treatment or withdrawal of consent after enrolment for personal reasons; failure to provide blood transfusion records in the previous 3 months; cumulative dose of the drug after enrolment was 30% lower than the total dose of the program. Finally, between August 2020 and December 2021, 185 patients were diagnosed and consecutively enrolled in this study when the parents signed the informed consent form (Flowchart in Figure 1). Of these patients, 15 were ineligible for efficacy and safety evaluation.

Thalidomide (Suzhou Changzheng-Xinkai Pharmaceutical Co. Ltd. (China)) was administered to patients at a dose of 2.5 to 4 mg/kg administered orally every day for 12 months until continuous transfusion-dependency or unacceptable toxicity occurred. Patients were then allowed to extend treatment beyond the investigator’s assessment of need and safety if criteria were met, including stable performance status and derived perceived clinical benefit. Patients were required to discontinue treatment if transfusion-dependency, peripheral nerve electrophysiological impairment after 12 months of therapy or other unacceptable toxicity occurred.

### Supportive care, transfusion and iron chelation

During treatment, patients received supportive care to reduce symptoms of adverse effects, including adenosine triphosphate for drowsiness, a high-fibre diet for constipation, vitamin D for arthralgia, vitamin B6 for numbness, antiallergic drugs for rash and liver-protectors. Vitamin B complexes and mecobalamin are prescribed for those with low peripheral nerve conduction velocity [21]. No prophylactic anti-thrombotic agent was used, except for patients with platelets >800 × 10^9^/L, who were treated with aspirin (orally, 3 mg/kg per day) and dipyridamole (orally, 1 mg/kg per day) (*n* = 4) to prevent thrombosis.

Blood transfusion was recommended to maintain haemoglobin above 90.0 g/L during treatment. Blood transfusion should be given if the haemoglobin level was below 90.0 g/L. The Hb level should not be lower than 80.0 g/L. Transfusion volume was recorded every 3 months according to the transfusion order in the medical record. Iron chelation therapy was continued if serum ferritin was above 1,000 ng/ml. One or two of deferiprone, deferoxamine, and deferasirox were recommended as iron chelators, depending on serum ferritin levels.

### Outcomes

Blood transfusion volume during the 12 consecutive weeks was compared with the blood transfusion volume during the 12 consecutive weeks of the previous follow-up cycle. When haemoglobin ≥90 g/L, maintaining transfusion independence was considered a major response, and when haemoglobin ≥90 g/L, reducing transfusion volume by ≥50% was considered a minor response. The overall response was defined as both major and minor response. Otherwise, it was defined as ineffective. The rate of discontinuation due to adverse events (AEs), effects on nerve conduction velocity, effects on height growth, and effects on menstruation in females were analyzed for the safety evaluation.

### The evaluation of AEs, height growth and nerve conduction velocity

Platelet, leucocytes, neutrophils, liver and renal function, and coagulation were monitored regularly. AEs and toxicity were graded according to the modified Common Terminology Criteria for Adverse Events (CTCAE) v5.0. According to the off-label medicine committee guidance, a dose reduction or withdrawal of thalidomide was performed if grade 3/4 AEs occurred.

Height growth velocity was calculated as the difference in height divided by the difference in months between two visits, and the annualized height growth velocity was 12 times the monthly height growth. The growth velocity (GV) was calculated using the formula (GV (cm/year) = (Height1-Height2) X 12/Δmonths). The interval between Height1 and Height2 (Δmonths) measurements should be at least 6 months. A single phase exponential decay was used to fit the curve for height. Standard deviation (SD) scores (SDS) of heights were calculated individually with reference to the standardized growth curves of height and weight of children and adolescents aged 0–18 years in China [22] at baseline and at the end of 12–18 months of treatment. In this study, height and weight were recorded at the outpatient clinic. To assess the effect of thalidomide on height growth, the height data of 108 patients were analyzed. Since the ages of the patients included were dispersed, the height growth velocity was calculated individually.

Peripheral nerve conduction velocity was measured in subjects when the treatment lasted for longer than 12 months. The motor conduction velocity of the left ulnar nerve, the left and right median nerves, the right tibial nerve, and the left and right peroneal nerves, and the sensory conduction velocity of the left ulnar nerve, the left and right median nerves, the right medial plantar nerve, the left and right superficial peroneal nerves and the right sural nerve were measured by electromyography (Shanghai Hanfei Medical Equipment Co., Ltd., China). The electrode was inserted into the muscle, and the biological current was amplified in the resting state of muscle contraction, and the motor conduction velocity and sensory conduction velocity were displayed on a cathode ray oscilloscope.

Pregnancy cannot be achieved while taking thalidomide and effective contraception or abstinence were recommended. The menstrual status of female patients was assessed by the menstrual history, which included age at first menstruation, cycle, last menstrual period, and answers were provided by the patients and family members during follow-up.

### Statistical analysis

Recruitment of 48 participants was planned so that a primary outcome overall response of ≥67% would provide 80% power with a one-sided α of 5% to reject a true rate of ≤50%. To provide sufficient numbers for the secondary outcome analysis, recruitment was planned to continue until 48 participants had completed nerve conduction velocity assessment.

Clinical data from both medical records and case report forms were collected prospectively from the date of initiation of prolonged treatment. The outpatient clinic and telephone follow-up were complementary access to the follow-up. In the extended follow-up, patients were excluded in they who could not provide a record of the transfusion volume before and after thalidomide use. Safety assessments were performed continuously during the outpatient visits. For patients who discontinued thalidomide to undergo allogeneic haematopoietic stem cell transplantation, response assessments (response or non-response) were performed at the time of discontinuation. Follow-up was updated on 31 December 2023.

Comparisons between two groups were calculated using Chi-square tests. Data were expressed as a median (range) or mean (95% confidence interval (CI)). The Kruskal-Wallis H test or one-way ANOVA analysis was used to identify factors that predicted efficacy. To compare variants between different groups, the Cochran-Mantel-Haenszel Chi-square test was conducted. The receiver-operating characteristic (ROC) curve analysis, Youden’s index and diagnostic accuracy were used to evaluate independent risk factors in predicting major responders, optimal cut-off values and the area under the ROC curve (AUC). Logistic regression was used to identify independent predictive factors for major response. Multiple imputation was used to account for missing values in the analysis of HbF concentration. Paired t-test was used to compare the difference between SDSs of height. SPSS 23.0 (SPSS Institute, Cary, NC) and GraphPad Prism 9.0 were used for statistical analysis. Significant *p* value was 0.05.

## Results

### Patients’ characteristics and efficacy evaluation

**Figure 2.**
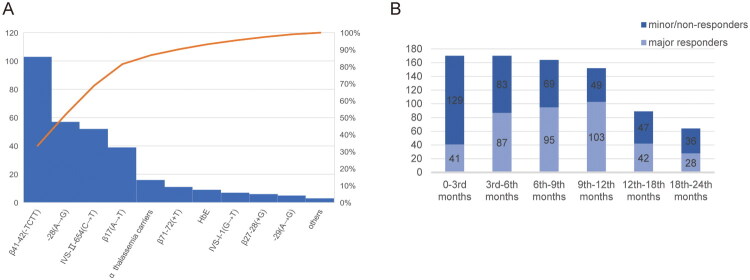
(A) Thalassaemia genotype disposition of the enrolled population. Thalassaemia gene variants of β41-42(-TCTT) (*n* = 103), IVS-II-654(C→T) (*n* = 52), −28(a→G) (*n* = 57), β17(a→T) (*n* = 39), HbE (*n* = 9), α-thalassaemia carriers (*n* = 16), β27-28(+G) (*n* = 6), β71-72(+T) (*n* = 11), IVS-I-1(G→T) (*n* = 7), −29(a→G) (*n* = 5) were detected in the included patients. (B) The efficacy of different stages of treatment.

One hundred and seventy patients were eligible, including 67 female and 103 male patients. The mean age was 10.3 (95% CI (10.0, 11.1)) years. Splenectomy was performed in 13 patients prior to enrolment of the current studies. Pre-thalidomide parameters were compared between major responders and minor/non-responders (Table 1). The effective dose of thalidomide ranged from 2.5 to 3.8 mg/kg/d. The genotype category includes β^0^/β^0^ (*n* = 61, 35.9%), β^0^/β^+^ (*n* = 71, 41.8%), and β^+^/β^+^ (*n* = 38, 22.4%). Genotype disposition and cumulative frequency are shown in Figure 2(A). Thalassaemia genotypes and response to thalidomide were not correlated. The overall response rate was 82.9% (141/170), including 102 (60.0%) major responders and 39 (22.9%) minor responders. When the patients were treated for up to 6 months, 102 (60.0%) maintained Hb ≥90 g/L for more than 3 months without blood transfusion. 39 (22.9%) patients had prolonged transfusion intervals or decreased transfusion volume with Hb ≥90 g/L. The efficacy of different stages of treatment is illustrated in Figure 2(B).

**Table 1.** The analysis of parameters for response differences.

	Major responders (*n* = 102)	Minor responders + non-responders (*n* = 68)	*p* value
Age, (years)	9.65 (9.10, 10.63)^*^	10.99 (10.34, 11.75)^*^	0.434*
Sex			
Male, *n*	62	41	0.537^‡^
Female, *n*	40	27	
Effective tolerable dosage of thalidomide, mean (95% CI) [g/(kg·d)]	3.64 (3.35, 3.93)^*^	3.51 (2.51, 4.51)^*^	0.913*
Genotype category			
β0/β0, *n* (%)	33	28	0.501^‡^
β0/β+, *n* (%)	45	26	
β+/β+, *n* (%)	24	14	
Transfusion interval			
≥28 days, *n* (%)	49	36	0.307^‡^
<28 days, *n* (%)	52	31	
Baseline HbF, (g/L)	8.87 (0.625, 77.1)^§^	3.49 (0, 16.7)^§^	<0.001^†^
HbF at month 3 of treatment, (g/L)	58.3 (51.6, 65.0)^*^	21.0 (0.9, 86.4)^§^	<0.001^†^

*p value from one-way ANOVA analysis of variance.

^†^*p* value from Kruskal-Wallis H test.

^‡^*p* value from Cochran-Mantel-Haenszel Chi-square test.

^*^Mean (95% confident interval).

^§^Percentiles, Q_50%_ (range).

### Predictive value of the HbF for probability of major responders

**Figure 3.**
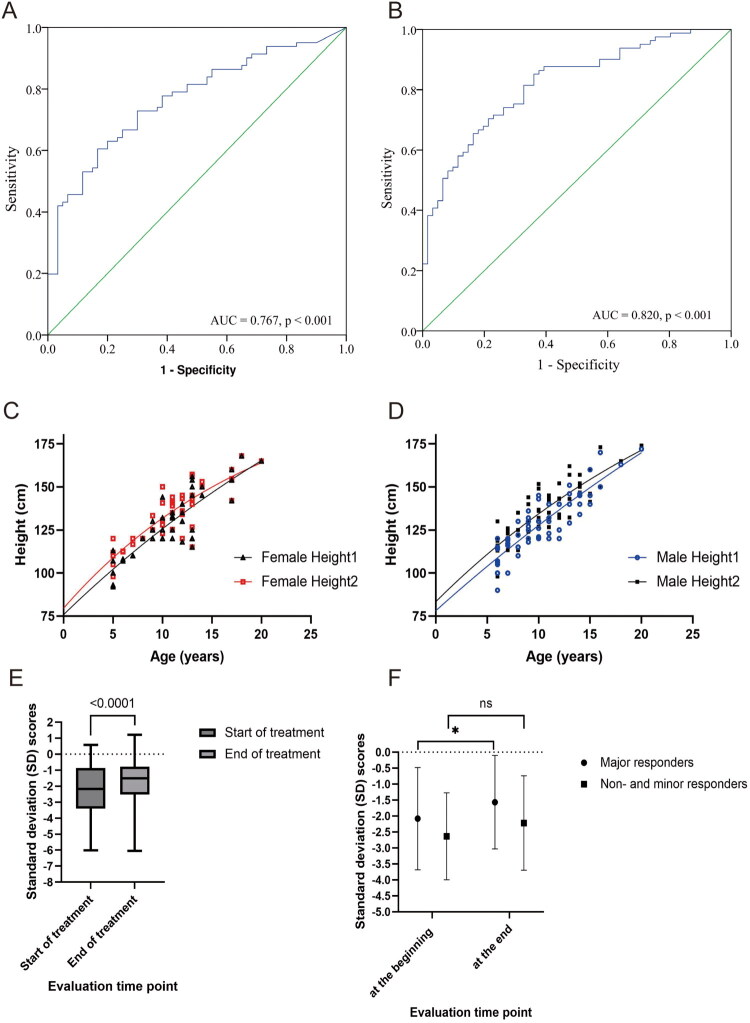
The long-term impact of thalidomide on children with β-thalassaemia. (A) The receiver-operating characteristic (ROC) analysis for haemoglobin F (HbF) concentration at baseline in predicting the probability of major responders. The area under the ROC curve (AUC) was 0.767 (*p* < 0.001). (B) The ROC analysis for HbF concentration at the third month in predicting the probability of major responders. The AUC was 0.820 (*p* < 0.001). (C) The baseline height (the black spots and black line, Female Height1) and the ending height (the red spots and red line, Female Height2) of the female group were distinct. (D) The baseline height (the blue spots and blue line, Male Height1) and the ending height (the black spots and black line, male Height2) of the male group were distinct. (E) Standard deviation scores (SDSs) of heights at baseline (−2.25, 95% CI (−2.58, −1.91)) and the end of 12 to 18 months of treatment (−1.76, 95% CI (−2.08, −1.44)) were significantly different (*p* < 0.001). (F) SDSs of the height of the major responder population at baseline (−2.08, 95% CI (−2.50, −1.66)) and the end of 12 to 18 months of treatment (−1.57, 95% CI (−1.95, −1.18)) were significantly different (*p < 0.001). SDSs of the height of non-responder and minor responder populations at baseline (−2.64, 95% CI (−3.20, −2.07)) and the end of 12 to 18 months of treatment (−2.22, 95% CI (−2.88, −1.69)) were not notably different (ns, *p* = 0.082).

In the extend treatment group, AUCs were 0.77 (95% confidence interval (0.69, 0.84), *p* < 0.001, for baseline HbF (Figure 3(A)) and 0.82 (95% confidence interval (0.75, 0.89), *p* < 0.001, for third-month HbF (Figure 3(B)). Youden’s index identified 26.89 g/L as the optimal cut-off for third-month HbF to predict a major response after prolonged treatment (Table 2). In logistic regression analysis, HbF concentration at month 3 is an independent predictor for major response (Table 3).

**Table 2.** Threshold analysis of the HbF concentration at different stages of thalidomide treatment for ROC of major responders.

Test	Best threshold	Specificity	Sensitivity	Accuracy	False positive rate	Odds Ratio (OR)	*p* for OR
Baseline HbF, (g/L)	7.285	0.933	0.605	0.703	0.067	1.044	<0.001
HbF at month 3 of treatment, (g/L)	26.89	0.852	0.639	0.761	0.147	1.135	0.007

Abbreviations: HbF, foetal haemoglobin; ROC, receiver-operating characteristic curve.

**Table 3.** Predictive factors for major responders towards thalidomide therapy.

Factors	*B*	*SE*	Wald *χ^2^*	Odds ratio (OR)	*p* for OR
A: Baseline HbF (≥7.285 g/L)	−1.388	0.875	2.516	0.250	0.113
B: HbF at month 3 of treatment (≥26.89 g/L)	−1.875	0.524	12.816	0.153	<0.001
A + B	0.246	0.997	0.061	1.278	0.805

Abbreviation: HbF, foetal haemoglobin.

### Safety

AEs were reported in 87 (51%) patients, of which 41 (24%) patients reported more than one AE. The AEs reported were dizziness and/or somnolence (*n* = 55, 32%), constipation (*n* = 15, 9%), abdominal pain and/or vomiting and/or nausea (*n* = 13, 8%), arthralgia (*n* = 8, 5%), oedema (*n* = 3, 2%), peripheral neuropathy (numbness of extremities) and/or headache (*n* = 8, 3.5%), skin rash (*n* = 4, 2.4%), chest tightness (*n* = 2, 1%), weight gain (*n* = 6, 4%), elevated bilirubin (*n* = 5, 3%), leukocytopenia (*n* = 22, 13%), neutropenia (*n* = 28, 16%), thrombocytosis (*n* = 33, 19%). The most common drug-related AEs of any grade were fatigue or lethargy (*n* = 49, 29%), constipation (*n* = 13, 8%), thrombocytosis (*n* = 15, 9%), abdominal pain (*n* = 12, 7%), and arthralgia (*n* = 8, 5%). Most of the symptoms were tolerable (*n* = 74, 44%). The majority of the patients’ symptoms resolved spontaneously without withdrawal, while some patients’ symptoms were alleviated with temporary dose reductions. Grade III/IV drug-related AEs were abdominal pain (*n* = 2), lethargy due to trauma (*n* = 1), haemolysis (*n* = 3), leukocytopenia and neutropenia (*n* = 2), deep vein thrombosis (*n* = 1), varicella (*n* = 1), facial nerve paralysis (*n* = 1), convulsion (*n* = 1), skin rash (*n* = 1), and nerve conduction disorders (*n* = 1). No treatment-related or disease -related mortality was observed. Fifteen patients (8%) discontinued treatment due to intolerable drug-related AEs; most commonly haemolysis (2%), acute hepatic failure (1%), and leukocytopenia and neutropenia (1%). One case of bacterial liver abscess and one case of emotional disturbance with somnambulism were reported during treatment. One case of chronic myeloid leukaemia, one case of Hodgkin’s lymphoma and one case of bacterial meningitis were reported after discontinuation.

There are also AEs that are hidden symptoms without the patient complaining. Seventy-three patients, who had received thalidomide for more than 12 months took part in the nerve conduction velocity test. All but one of the patients tested had no symptoms of numbness in the extremities. Thirty-nine patients had impaired peripheral nerve conduction velocity, including 12 with polyneuropathy, 26 with motor neuropathy, and 2 with sensory neuropathy. The incidence of electrophysiological neuropathy was not related to response (*p* = 0.56). A decrease in peripheral nerve conduction velocity is correlated with intolerable toxicity (*p* = 0.044), but not with lethargy (*p* = 0.372), constipation (*p* = 0.644), numbness in the extremities (*p* = 0.469), abdominal pain (*p* = 0.278), or arthralgia (*p* = 0.644). Age, sex, and thalidomide dose did not differ between patients with and without peripheral nerve conduction velocity impairment. Three patients had follow-up nerve conduction studies, and none of them showed an improvement in their nerve conduction velocity after a 3-month break in thalidomide therapy. Although there was no electrophysiological improvement in these patients, the sensory and motor nerve action potential amplitudes remained stable.

Height growth was greater than or equal to 10 cm in 22 patients (20%), less than or equal to 5 cm in 56 patients (52%), and greater than 5 cm but less than 10 cm in 30 patients (28%). Five patients complained of height growth retardation. Both female and male populations both showed an increase in height when comparing baseline and final height (Figure 3(C,D)). The SDS of height at baseline (−2.25, 95% CI (−2.58, −1.91)) and at the end of 12 to 18 months of treatment (−1.76, 95% CI (−2.08, −1.44)) were significantly different (*p* < 0.001) (Figure 3(E)). The SDS of height at baseline (−2.08, 95% CI (−2.50, −1.66)) and at the end of 12 to 18 months of treatment (−1.57, 95% CI (−1.95, −1.18)) in major responders were significantly different (*p* < 0.001) (Figure 3(F)), while the changes in SDS in the non-responders and minor responders were not significantly different (*p* = 0.082) (Figure 3(F)). 64% of patients showed improvement in SDS after more than 1 year of treatment.

Of the 19 females aged 13 years or older, 13 patients menstruated during the treatment, of whom 6 had regular menstruation. Of the females younger than 13 years (*n* = 48), 40 did not menstruate, 7 had regular menstruation, and 1 had irregular menstruation. Data on male testicular volume data were not collected.

## Discussion

The efficacy and safety of long-term thalidomide in children with TDT are presented in this extended large-scale follow-up data analysis. To our knowledge, this is the longest follow-up report of long-term thalidomide treatment in children with TDT. We have several important findings. Firstly, the overall response rate is as high as with short-term therapy. Second, the assessment of HbF concentration at month 3 predicts the chances of benefit when treatment is prolonged. At the same time, height growth is improved in responders, but about half of the children with TDT develop electrophysiological impairment in the peripheral nerves after prolonged therapy.

In a previous study, increasing the dose did not increase the response rate [6]. To improve response, increasing the dose up to 3.5 to 4.0 mg/kg/d might help some non-responders respond better or become transfusion-independent. However, this would increase adverse events. Long-term observation provides more information about the rare side effects of thalidomide treatment for TDT. Dizziness and/or somnolence led to the common phenomenon of nodding off in school and one case of trauma. As impaired peripheral neuron function and electrophysiological abnormalities have been widely reported to be associated with cumulative doses of thalidomide [11,17,23], we propose to examine nerve conduction velocity at the end of the 12th month of thalidomide therapy and found that more than half of the patients had impaired peripheral nerve conduction velocity without physical symptoms. At the doses of 2.5 to 3.8 mg/kg/d, the nerve conduction velocity results were similar to those reported in the literature, suggesting that the pause is necessary to protect nerve function in the TDT patients who intend to prolong treatment.

Height growth retardation is one of the most common complications associated with endocrine dysfunction in TDT, particularly in those with inadequate transfusion [24,25]. Given the teratogenic effect of thalidomide [26], whether it affects height growth has been a major concern for long-term use. In this study, we collected the height data from participants and found that half of the patients grew less than or equal to 5 cm in 1 year, indicating growth retardation prior to thalidomide treatment. SDS was significantly improved in major responders. Iron load was not correlated with growth rate during thalidomide therapy, while the endocrine regulation was not studied, suggesting that further studies are needed to understand this area. Thalidomide can cause reversible ovarian dysfunction leading to amenorrhoea [27,28]. We recorded the menstrual status of some female patients. Most of the patients who had menarche are able to maintain normal menstruation. However, in patients with abnormal menarche, we cannot exclude the important factor of thalassaemia-related iron overload.

There is another important piece of information about late-onset adverse events. As a traditional immunomodulator, thalidomide can regulate and inhibit the immune system after long-term use [11,18]. In this study, tumours and late-onset infections were reported in those who had discontinued thalidomide therapy, and it is not clear whether there is a correlation with the effect of immunosuppression.

The baseline HbF and post-treatment HbF concentrations represent the activity of *HBG* expression. According to the previous report, HbF concentration after 3 months of treatment is a potential predictor of responders. The ROC curves showed that third-month HbF performed well in predicting major response [6]. In the analysis of the long-term follow-up data, we find another cut-off value of HbF concentration, 26.89 g/L, for a major response in long-term treatment. This is different from the previous finding in the short-term follow-up [6]. Our large data suggest that we can try to extend the observation for up to 6 months based on the early phase HbF concentration. Even if the HbF concentration is lower than 47.298 g/L in the third month of treatment [6], it is possible that the patient, with an HbF concentration higher or equal to 26.89 g/L in the third month, could continue thalidomide and become transfusion independent in a prolonged therapy. Non β0/β0 or certain genomic combinations (like βCD41-42/β-28, CD41-42/βIVS-II-654 genotypes) and certain HBS1L-MYB genotypes have been reported to be associated with good responses [1,4]. However, the use of HbF concentration to predict response is more practical and operational than genotype detection.

Numerous adverse effects of thalidomide were shown in the study, and how to balance the benefits and risks has become a concern for parents of patients and clinicians. Recently, it has been reported that in developing Asian countries such as China, thalassaemia patients often have to wait longer for blood transfusions due to high population density and a shortage of blood sources [29,30]. The use of inexpensive thalidomide, which allows patients to stop transfusion for a period of time or extend the interval between transfusions, is more effective and acceptable in reducing the amount of blood transfused than the more expensive luspatercept [31]. Mitapivat is not yet available and has not been used in children. Unlike luspatercept, thalidomide has shown some inhibition of bone marrow hyperproliferation, no adverse effects on extramedullary haematopoiesis or hypertension, and has been shown to reduce hepatosplenomegaly [31]. The current study also found an improvement in height in children, which has not been reported for luspatercept and mitapivat.

In conclusion, this is the longest follow-up of children with TDT treated with thalidomide reported to date. Thalidomide shows a high ORR and durable response. Overall, the results of this study suggest that the use of thalidomide may provide long-term benefits in terms of transfusion independence and improved height growth in children with TDT. We recommend evaluation of HbF concentration at month 3 to predict the likelihood of benefit with prolonged treatment. The exploratory analysis presented here further supports the protocol of continuing thalidomide therapy for up to 1 year, while monitoring neuropathy during long-term therapy. Neuropathy and unexpected adverse events prevent the long-term use of thalidomide in children with thalassaemia. Unfortunately, there is no better way to reduce the adverse effects of this therapy than to discontinue it. Further clinical observation is needed to determine how long it should be before thalidomide is started again.

## Supplementary Material

CONSORT 2010 Checklist.doc

## Data Availability

The datasets generated and/or analyzed during the current study are available from the corresponding author upon reasonable request.

## References

[1] Yang WJ, Kang QP, Zhou Q, et al. Clinical efficacy of thalidomide for various genotypes of beta thalassemia. BMC Med Genomics. 2024;17(1):191. doi:10.1186/s12920-024-01963-y. 39026312 PMC11264728

[2] Chen J, Zhu W, Cai N, et al. Thalidomide induces haematologic responses in patients with beta-thalassaemia. Eur J Haematol. 2017;99(5):437–441. doi:10.1111/ejh.12955. 28850716

[3] Ren Q, Zhou YL, Wang L, et al. Clinical trial on the effects of thalidomide on hemoglobin synthesis in patients with moderate thalassemia intermedia. Ann Hematol. 2018;97(10):1933–1939. doi:10.1007/s00277-018-3395-5. 29931453

[4] Chen JM, Zhu WJ, Liu J, et al. Safety and efficacy of thalidomide in patients with transfusion-dependent beta-thalassemia: a randomized clinical trial. Signal Transduct Target Ther. 2021;6(1):405. doi:10.1038/s41392-021-00811-0. 34795208 PMC8602273

[5] Yang K, Wu Y, Zhou Y, et al. Thalidomide for patients with beta-thalassemia: a multicenter experience. Mediterr J Hematol Infect Dis. 2020;12(1):e2020021. doi:10.4084/MJHID.2020.021. 32395210 PMC7202343

[6] Li X, Hu S, Liu Y, et al. Efficacy of thalidomide treatment in children with transfusion dependent beta-thalassemia: a retrospective clinical study. Front Pharmacol. 2021;12:722502. doi:10.3389/fphar.2021.722502. 34456732 PMC8397440

[7] Lu Y, Wei Z, Yang G, et al. Investigating the efficacy and safety of thalidomide for treating patients with ss-thalassemia: a meta-analysis. Front Pharmacol. 2021;12:814302. doi:10.3389/fphar.2021.814302. 35087410 PMC8786914

[8] Ali Z, Rehman IM, Rani IU, et al. Long-term clinical efficacy and safety of thalidomide in patients with transfusion-dependent β-thalassemia: results from Thal-Thalido study. Sci Rep. 2023;13(1):13592. doi:10.1038/s41598-023-40849-4. 37604857 PMC10442319

[9] Chandra, Jagdish, Parakh, Nupur, Singh, Neha, et al. Efficacy and safety of thalidomide in patients with transfusion-dependent thalassemia. Indian Pediatr. 2021;58(7): 611–616. doi:10.1007/s13312-021-2254-y. 34315832

[10] Che J, Luo T, Huang L, et al. Magnetic resonance imaging quantification of the liver iron burden and volume changes following treatment with thalidomide in patients with transfusion-dependent ss-thalassemia. Front Pharmacol. 2022;13:810668. doi:10.3389/fphar.2022.810668. 35250561 PMC8894715

[11] Lazzerini M, Martelossi S, Magazzù G, et al. Effect of thalidomide on clinical remission in children and adolescents with refractory Crohn disease: a randomized clinical trial. JAMA. 2013;310(20):2164–2173. doi:10.1001/jama.2013.280777. 24281461

[12] Bramuzzo M, Stocco G, Montico M, et al. Risk factors and outcomes of thalidomide-induced peripheral neuropathy in a pediatric inflammatory bowel disease cohort. Inflamm Bowel Dis. 2017;23(10):1810–1816. doi:10.1097/MIB.0000000000001195. 28817461

[13] van Toorn R, Zaharie SD, Seddon JA, et al. The use of thalidomide to treat children with tuberculosis meningitis: a review. Tuberculosis (Edinb). 2021;130:102125. doi:10.1016/j.tube.2021.102125. 34500217

[14] Soper JR, Bonar SF, O’Sullivan DJ, et al. Thalidomide and neurotrophism. Skeletal Radiol. 2019;48(4):517–525. doi:10.1007/s00256-018-3086-2. 30341712 PMC6394469

[15] Yang CS, Kim C, Antaya RJ. Review of thalidomide use in the pediatric population. J Am Acad Dermatol. 2015;72(4):703–711. doi:10.1016/j.jaad.2015.01.002. 25617013

[16] Gunaseelan S, Prakash A. Thalidomide-induced stroke in a child with thalassemia major. J Pediatr Hematol Oncol. 2017;39(8):e519–e520. doi:10.1097/MPH.0000000000000860. 28538510

[17] Liew WK, Pacak CA, Visyak N, et al. Longitudinal patterns of thalidomide neuropathy in children and adolescents. J Pediatr. 2016;178:227–232. doi:10.1016/j.jpeds.2016.07.040. 27567409

[18] Chandra J, Safety A, Primary C, et al. Reply. Indian Pediatr. 2021;58(11):1100–1101. doi:10.1007/s13312-021-2386-0. 34837374

[19] Grech L, Sultana J, Borg K, et al. Drug safety in thalassemia: lessons from the present and directions for the future. Expert Opin Drug Saf. 2021;20(8):937–947. doi:10.1080/14740338.2021.1919081. 33877003

[20] Jian X, Liu X, Peng W, et al. Long-term efficacy and safety of thalidomide treatment in children with β -thalassemia major. Pediatr Blood Cancer. 2023;70(9):e30391. 37114720 10.1002/pbc.30391

[21] Sawangjit R, Thongphui S, Chaichompu W, et al. Efficacy and safety of mecobalamin on peripheral neuropathy: a systematic review and meta-analysis of randomized controlled trials. J Altern Complement Med. 2020;26(12):1117–1129. doi:10.1089/acm.2020.0068. 32716261

[22] Li H, Ji CY, Zong XN, et al. Height and weight standardized growth charts for Chinese children and adolescents aged 0 to 18 years. Zhonghua Er Ke Za Zhi. 2009;47(7):487–492. 19951507

[23] Chaudhry V, Cornblath DR, Corse A, et al. Thalidomide-induced neuropathy. Neurology. 2002;59(12):1872–1875. doi:10.1212/01.wnl.0000037480.59194.85. 12499476

[24] Arab-Zozani M, Kheyrandish S, Rastgar A, et al. A systematic review and meta-analysis of stature growth complications in beta-thalassemia major patients. Ann Glob Health. 2021;87(1):48. doi:10.5334/aogh.3184. 34164261 PMC8194969

[25] Chuansumrit A, Sirachainan N, Kitpoka P, et al. The effect of blood transfusion on growth of patients with Hb E/beta-thalassemia. Hemoglobin. 2019;43(4-5):264–272. doi:10.1080/03630269.2019.1692863. 31760834

[26] Gao S, Wang S, Fan R, et al. Recent advances in the molecular mechanism of thalidomide teratogenicity. Biomed Pharmacother. 2020;127:110114. doi:10.1016/j.biopha.2020.110114. 32304852

[27] Singha A, Mukhopadhyay P, Ghosh S. Thalidomide-induced primary amenorrhea in a patient with HbE/beta-thalassemia. JCEM Case Rep. 2023;1(3):luad057. 37908579 10.1210/jcemcr/luad057 PMC10580460

[28] Frances C, Gompel EKS, Bécherel A, et al. Transient secondary amenorrhea in women treated by thalidomide. Eur J Dermatol. 2002;12(1):63–65. 11809598

[29] Wang WD, Hu F, Zhou DH, et al. Thalassaemia in China. Blood Rev. 2023;60:101074. doi:10.1016/j.blre.2023.101074. 36963988

[30] Chen P, Lin WX, Li SQ. THALASSEMIA in ASIA 2021: thalassemia in Guangxi Province, People’s Republic of China. Hemoglobin. 2022;46(1):33–35. doi:10.1080/03630269.2021.2008960. 35950576

[31] Cappellini MD, Viprakasit V, Taher AT, et al. A phase 3 trial of luspatercept in patients with transfusion-dependent beta-thalassemia. N Engl J Med. 2020;382(13):1219–1231. doi:10.1056/NEJMoa1910182. 32212518

